# Osteomyelitis of the jaw treated with gentamicin slow releasing beads – a Case report

**DOI:** 10.1186/1471-2334-14-S7-P49

**Published:** 2014-10-15

**Authors:** Iulian Filipov, Lucian Chirilă, Cristian Rotaru, Mihai Săndulescu

**Affiliations:** 1 “Dan Theodorescu” Clinical Hospital of Oral and Maxillo-Facial Surgery, Bucharest, Romania; 2 Carol Davila University of Medicine and Pharmacy, Bucharest, Romania; 3 Dental Concept Studio, Bucharest, Romania

## Background

Osteomyelitis is the infection of the bone. The local infectious process can begin as a consequence of a direct injury to the bone with direct insemination of bacteria, it can spread from nearby tissues or bacteria can travel through the bloodstream from distant infectious sites in the body. The jaws are commonly affected sites, mostly by direct insemination or spreading from nearby tissues. The most common bacteria involved is *Staphylococcus aureus*. Regardless of the way the bone is infected, the end result is the same, with the development of an acute response with fever, pain, pus formation, swelling and if the mandibular bone is affected a neurological complication with anesthesia can occur. The aim of this case report is to present a complementary local treatment and its clinical outcomes.

## Case report

We present the case of a male patient with mandibular osteomyelitis treated with radical surgical debridement and slow releasing gentamicin beads. The therapy had a good outcome with bone healing after 30 days and no signs of infection. The patient underwent a second surgery after 2 years for the removal of the beads, which were found to be fully integrated in the newly formed bone.

## Conclusion

The treatment for osteomyelitis can be sometimes very inconvenient for the patient, as the treatment usually includes hospitalization with surgical intervention and intravenous antibiotics. The use of local slow releasing gentamicin beads is a complementary and not alternative treatment. We achieved good results with this technique that lead to bone healing with the full integration of the beads.

